# Correction: Dispersion stability and tribological properties of additives introduced by ultrasonic and microwave assisted ball milling in oil

**DOI:** 10.1039/d3ra90006a

**Published:** 2023-01-18

**Authors:** Siyuan Wang, Ding Chen, Yaotong Chen, Kaiji Zhu

**Affiliations:** a State Key Laboratory of Advanced Design and Manufacturing for Vehicle Body, Hunan University 410082 Changsha China chending@hnu.edu.cn; b College of Materials Science and Engineering, Hunan University 410082 Changsha China

## Abstract

Correction for ‘Dispersion stability and tribological properties of additives introduced by ultrasonic and microwave assisted ball milling in oil’ by Siyuan Wang *et al.*, *RSC Adv.*, 2020, **10**, 25177–25185, https://doi.org/10.1039/D0RA03414B.

The authors regret that an incorrect version of Fig. 15b was included in the original article. The correct version of Fig. 15 is presented below.

**Fig. 15:**
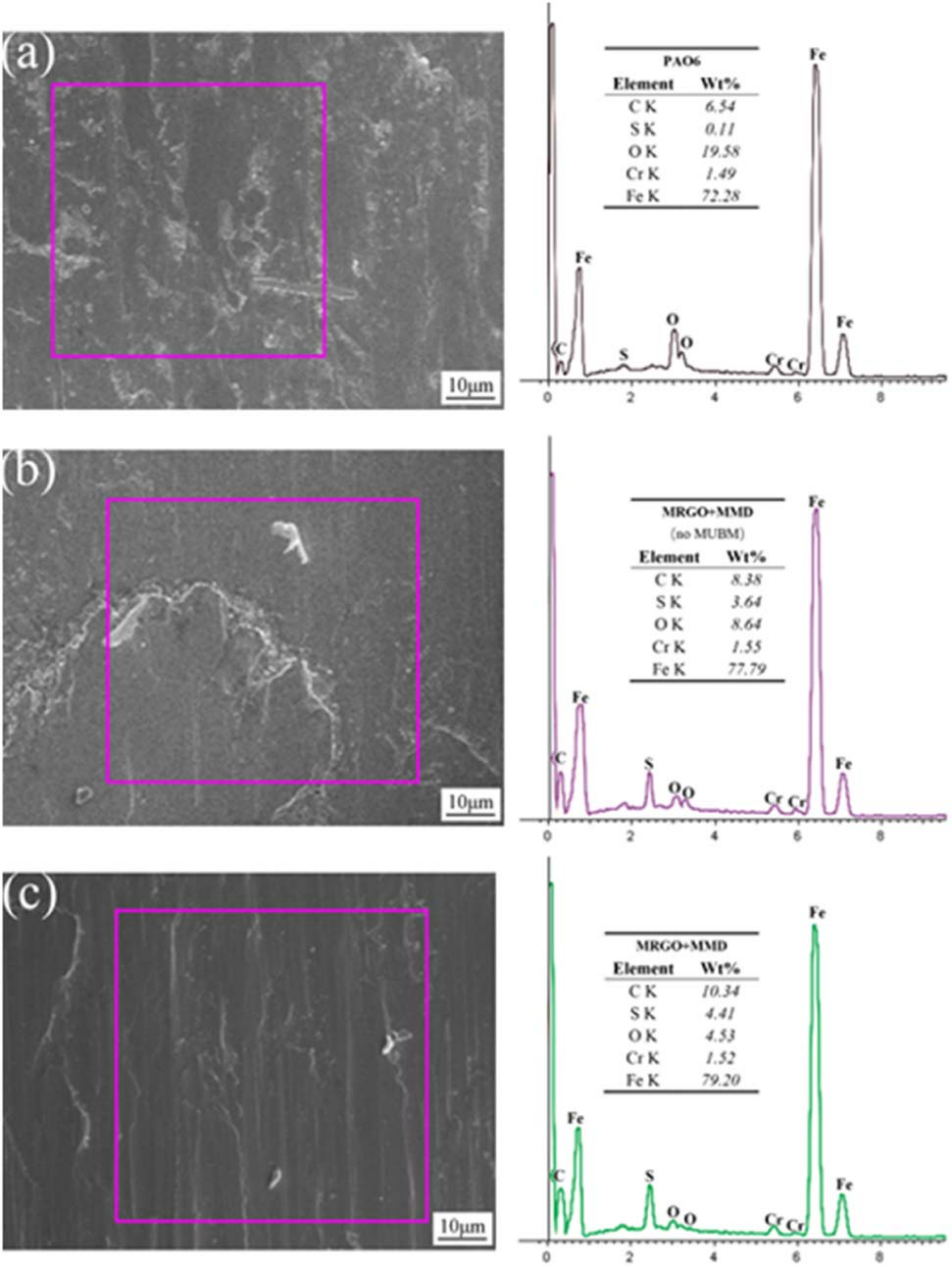
SEM and EDS images of the friction surface are lubricated with (a) the base oil, (b) the lubricating oil without UMBM treatment, and (c) the lubricating oil by UMBM treatment.

An independent expert has viewed the corrected images and has concluded that they are consistent with the discussions and conclusions presented.

The Royal Society of Chemistry apologises for these errors and any consequent inconvenience to authors and readers.

## Supplementary Material

